# Supplementary material for: A Likelihood Approach for Real-Time Calibration of Stochastic Compartmental Epidemic Models
Source: PLoS Comput Biol. 2017 Jan 17;13(1):e1005257. doi: 10.1371/journal.pcbi.1005257 (PMC5240920; doi:10.1371/journal.pcbi.1005257)
Supplement: S1 File — (TAR.GZ) [file pcbi.1005257.s014.tar.gz › HSPH_Online-SI-Revision/Additions/EnKF08/n100-extreme_noisy/n100-extreme_noisy_EnKF2_table8.pdf]

1	newly 3 weeks spec	526.162	2975.11	{92., 8027.}	507.782	0.604212	newly 3 weeks cumul	2501.43	14776.5	{1224., 33890.}	511.257	0.527271	newly 5 weeks spec	280.291	1612.48	{3., 6619.}	546.556	0.846149	newly 5 weeks cumul	3116.21	18535.5	{1319., 52725.}	518.315	0.53875	newly 46 weeks cumul	6824.91	24950.3	{1323., 79103.}	313.726	0.505327
2	newly 3 weeks spec	761.	3567.57	{63., 13987.}	416.269	0.662024	newly 3 weeks cumul	3077.	15650.	{1653., 38703.}	431.355	0.516487	newly 5 weeks spec	441.	2345.87	{0., 9393.}	490.887	0.950209	newly 5 weeks cumul	4068.	20981.7	{2313., 61821.}	438.291	0.526278	newly 42 weeks cumul	5247.	28688.4	{3385., 89244.}	467.284	0.544607
3	newly 3 weeks spec	277.	3134.26	{302., 6544.}	1036.83	0.791648	newly 3 weeks cumul	1321.	16526.5	{3348., 28986.}	1151.27	0.846646	newly 5 weeks spec	122.	1635.04	{28., 2822.}	1265.39	0.857211	newly 5 weeks cumul	1607.	20303.5	{3567., 37611.}	1163.76	0.866702	newly 47 weeks cumul	1853.	27396.7	{4396., 86341.}	1378.97	0.923258
4	newly 3 weeks spec	75.	401.89	{0., 619.}	492.227	0.969741	newly 3 weeks cumul	495.	3594.69	{129., 3001.}	654.382	0.683986	newly 5 weeks spec	26.	179.59	{0., 360.}	668.192	1.0758	newly 5 weeks cumul	564.	4036.04	{149., 3574.}	643.436	0.685362	newly 51 weeks cumul	589.	4592.74	{162., 4518.}	707.056	0.713144
5	newly 3 weeks spec	383.	3728.81	{603., 8978.}	880.102	0.779499	newly 3 weeks cumul	1884.	12796.	{3218., 20996.}	586.645	0.671122	newly 5 weeks spec	164.	2566.24	{327., 9140.}	1470.4	0.928425	newly 5 weeks cumul	2324.	18475.1	{3982., 41984.}	701.876	0.722523	newly 48 weeks cumul	2679.	27836.8	{5108., 89999.}	944.931	0.799595
6	newly 3 weeks spec	620.	3296.57	{442., 8352.}	443.676	0.586191	newly 3 weeks cumul	2591.	20302.9	{3073., 54957.}	686.583	0.698027	newly 5 weeks spec	303.	1227.32	{121., 2210.}	339.432	0.598256	newly 5 weeks cumul	3314.	23474.1	{3558., 62463.}	612.545	0.672448	newly 46 weeks cumul	4005.	26040.3	{3787., 64410.}	556.164	0.651598
7	newly 3 weeks spec	425.	2439.05	{62., 5694.}	525.922	0.711449	newly 3 weeks cumul	2093.	10687.	{308., 28461.}	450.416	0.580723	newly 5 weeks spec	238.	790.04	{1., 1068.}	296.983	0.862789	newly 5 weeks cumul	2658.	12852.2	{394., 33925.}	423.765	0.578615	newly 44 weeks cumul	3121.	14430.	{449., 36338.}	401.374	0.587039
8	newly 3 weeks spec	611.	423.94	{8., 671.}	103.159	0.930003	newly 3 weeks cumul	2634.	4296.39	{395., 14595.}	156.922	0.502061	newly 5 weeks spec	315.	105.74	{0., 266.}	88.2159	1.59926	newly 5 weeks cumul	3371.	4604.28	{435., 15590.}	139.952	0.546982	newly 46 weeks cumul	4133.	4809.42	{451., 15595.}	126.921	0.592796
9	newly 3 weeks spec	509.	950.1	{95., 2125.}	122.912	0.356821	newly 3 weeks cumul	2442.	19668.7	{5548., 56318.}	715.919	0.779514	newly 5 weeks spec	256.	120.69	{1., 271.}	96.6914	1.10646	newly 5 weeks cumul	3053.	20093.6	{5684., 57381.}	568.704	0.704091	newly 46 weeks cumul	3616.	20221.4	{5687., 57443.}	469.97	0.641648
10	newly 3 weeks spec	32.	2533.01	{73., 9128.}	7818.97	1.3569	newly 3 weeks cumul	165.	9593.19	{413., 20420.}	5715.98	1.34458	newly 5 weeks spec	7.	1941.89	{5., 5260.}	27657.6	1.74541	newly 5 weeks cumul	184.	13716.7	{506., 43228.}	7356.6	1.43408	newly 50 weeks cumul	192.	24138.8	{601., 73581.}	12474.2	1.5945
11	newly 3 weeks spec	582.	795.03	{40., 2897.}	108.881	0.478342	newly 3 weeks cumul	2565.	5725.39	{628., 25889.}	162.74	0.350081	newly 5 weeks spec	318.	207.07	{3., 645.}	74.9528	0.735081	newly 5 weeks cumul	3307.	6316.18	{643., 27191.}	141.781	0.376517	newly 44 weeks cumul	4044.	6702.56	{643., 27356.}	127.405	0.408668
12	newly 3 weeks spec	488.	5958.12	{1129., 13925.}	1121.49	0.858834	newly 3 weeks cumul	2113.	35073.5	{7666., 88678.}	1560.6	1.04032	newly 5 weeks spec	223.	1198.34	{91., 2113.}	457.731	0.605235	newly 5 weeks cumul	2630.	38885.8	{9700., 93409.}	1379.17	1.0025	newly 45 weeks cumul	3083.	40405.	{10849., 94855.}	1211.15	0.960449
13	newly 3 weeks spec	1177.	2927.51	{68., 9943.}	227.382	0.662113	newly 3 weeks cumul	4644.	26580.9	{1008., 100371.}	500.845	0.536906	newly 5 weeks spec	679.	533.81	{0., 1433.}	119.73	1.26498	newly 5 weeks cumul	6187.	28305.8	{1143., 103098.}	392.503	0.508859	newly 43 weeks cumul	8302.	28815.4	{1201., 103227.}	292.258	0.483982
14	newly 3 weeks spec	9.	2150.87	{0., 6810.}	23837.7	1.98204	newly 3 weeks cumul	64.	13663.	{1084., 46474.}	21248.4	1.99073	newly 5 weeks spec	5.	1693.34	{0., 8163.}	33812.8	2.01624	newly 5 weeks cumul	74.	17354.7	{1427., 58629.}	23352.3	2.03108	newly 48 weeks cumul	86.	22046.4	{1831., 89412.}	25535.4	2.05312
15	newly 3 weeks spec	73.	3118.54	{41., 16257.}	4189.84	0.91458	newly 3 weeks cumul	382.	8812.4	{804., 35164.}	2215.98	0.856001	newly 5 weeks spec	19.	1488.96	{3., 4423.}	7768.53	1.10593	newly 5 weeks cumul	449.	12720.9	{952., 66705.}	2742.35	0.880905	newly 48 weeks cumul	476.	14339.8	{965., 74825.}	2921.79	0.911863
16	newly 3 weeks spec	111.	3158.04	{176., 9900.}	2750.04	1.01255	newly 3 weeks cumul	653.	13218.3	{1799., 39867.}	1924.63	0.95888	newly 5 weeks spec	31.	1414.93	{18., 5220.}	4482.03	1.19869	newly 5 weeks cumul	748.	16631.6	{1829., 48557.}	2123.48	0.99459	newly 48 weeks cumul	812.	21724.7	{2366., 84206.}	2575.46	1.04798
17	newly 3 weeks spec	848.	1819.3	{428., 3081.}	145.62	0.274102	newly 3 weeks cumul	3379.	8542.23	{3565., 14711.}	160.043	0.275422	newly 5 weeks spec	459.	856.34	{104., 1287.}	152.898	0.416663	newly 5 weeks cumul	4480.	10632.5	{3998., 18007.}	147.138	0.247539	newly 43 weeks cumul	5685.	12983.4	{4182., 20620.}	145.804	0.240531
18	newly 3 weeks spec	371.	1031.05	{89., 2935.}	230.423	0.463158	newly 3 weeks cumul	1789.	15476.5	{4035., 27557.}	769.368	0.638687	newly 5 weeks spec	168.	93.1	{2., 219.}	82.9643	0.935229	newly 5 weeks cumul	2207.	15866.2	{4046., 28793.}	623.978	0.567342	newly 44 weeks cumul	2601.	15916.7	{4046., 28935.}	517.801	0.505907
19	newly 3 weeks spec	522.	1983.44	{358., 4314.}	293.824	0.336661	newly 3 weeks cumul	2300.	8708.66	{3119., 11256.}	285.627	0.394636	newly 5 weeks spec	225.	990.63	{58., 2425.}	381.471	0.44853	newly 5 weeks cumul	2858.	11110.9	{3746., 22339.}	295.553	0.385577	newly 46 weeks cumul	3344.	13768.5	{4042., 31952.}	317.977	0.387619
20	newly 3 weeks spec	31.	2548.3	{63., 9390.}	8123.1	1.36461	newly 3 weeks cumul	168.	12108.1	{316., 35434.}	7107.61	1.40601	newly 5 weeks spec	4.	808.81	{18., 2755.}	20123.3	1.71528	newly 5 weeks cumul	185.	14314.8	{360., 47006.}	7637.89	1.43859	newly 51 weeks cumul	187.	16276.8	{402., 57765.}	8604.18	1.48982
21	newly 3 weeks spec	203.	2096.1	{71., 7670.}	950.305	0.866659	newly 3 weeks cumul	1027.	7075.24	{948., 17245.}	604.896	0.787527	newly 5 weeks spec	76.	1907.26	{9., 9157.}	2435.11	1.08852	newly 5 weeks cumul	1233.	11014.6	{992., 35577.}	809.774	0.849924	newly 48 weeks cumul	1341.	20493.	{994., 91492.}	1444.82	0.974824
22	newly 3 weeks spec	138.	455.38	{0., 1238.}	323.565	1.14627	newly 3 weeks cumul	788.	8197.86	{1844., 17032.}	944.528	0.782608	newly 5 weeks spec	76.	158.18	{0., 251.}	254.737	1.78067	newly 5 weeks cumul	953.	8604.16	{2246., 17284.}	807.454	0.729663	newly 49 weeks cumul	1046.	9178.13	{2557., 17895.}	782.292	0.71737
23	newly 3 weeks spec	646.	3967.19	{202., 13768.}	558.116	0.519404	newly 3 weeks cumul	2717.	24734.	{3848., 79139.}	810.342	0.714866	newly 5 weeks spec	343.	385.06	{6., 1112.}	141.493	0.954481	newly 5 weeks cumul	3563.	26415.7	{3935., 88649.}	641.634	0.615253	newly 42 weeks cumul	4442.	26580.3	{3935., 89539.}	502.228	0.538726
24	newly 3 weeks spec	888.	1304.27	{0., 337.}	214.596	1.67319	newly 3 weeks cumul	3507.	5920.06	{511., 2061.}	199.054	0.637295	newly 5 weeks spec	441.	392.67	{0., 111.}	164.02	2.33705	newly 5 weeks cumul	4589.	7079.19	{558., 2337.}	193.938	0.715291	newly 42 weeks cumul	5895.	8056.49	{594., 2475.}	183.989	0.793607
25	newly 3 weeks spec	135.	2678.31	{199., 9992.}	1891.92	0.82379	newly 3 weeks cumul	801.	8682.89	{1413., 30567.}	991.527	0.717148	newly 5 weeks spec	46.	1538.92	{33., 4269.}	3259.22	0.944663	newly 5 weeks cumul	936.	12404.6	{1699., 48905.}	1232.73	0.760995	newly 45 weeks cumul	1018.	15742.7	{1704., 56219.}	1453.79	0.811877
26	newly 3 weeks spec	1048.	567.55	{51., 1608.}	83.8235	0.750959	newly 3 weeks cumul	4173.	6923.97	{1502., 11971.}	109.394	0.295561	newly 5 weeks spec	644.	141.74	{0., 327.}	89.618	1.63263	newly 5 weeks cumul	5651.	7332.78	{1534., 14621.}	94.8413	0.344182	newly 43 weeks cumul	7533.	7495.24	{1534., 16113.}	86.8862	0.416892
27	newly 3 weeks spec	806.	4450.77	{789., 12794.}	457.994	0.490395	newly 3 weeks cumul	3332.	17456.1	{3453., 61601.}	425.914	0.497385	newly 5 weeks spec	451.	2068.7	{251., 5167.}	380.878	0.505647	newly 5 weeks cumul	4401.	22618.1	{4918., 79019.}	416.563	0.489759	newly 44 weeks cumul	5811.	26548.8	{6080., 91305.}	360.645	0.458676
28	newly 3 weeks spec	568.	1882.41	{89., 2388.}	273.79	0.548311	newly 3 weeks cumul	2482.	11289.7	{1974., 10763.}	369.409	0.465813	newly 5 weeks spec	266.	923.23	{6., 1697.}	306.816	0.783646	newly 5 weeks cumul	3132.	13485.5	{1985., 11874.}	348.344	0.474803	newly 48 weeks cumul	3853.	16347.7	{2150., 19646.}	344.651	0.49534
29	newly 3 weeks spec	769.	552.59	{38., 1617.}	89.1847	0.612455	newly 3 weeks cumul	3241.	8493.01	{3626., 8404.}	165.913	0.216473	newly 5 weeks spec	412.	81.85	{0., 179.}	88.0558	1.46615	newly 5 weeks cumul	4213.	8767.98	{3717., 10303.}	119.512	0.157667	newly 42 weeks cumul	5339.	8846.03	{3720., 11379.}	105.32	0.206885
30	newly 3 weeks spec	278.	782.91	{151., 1417.}	198.054	0.38661	newly 3 weeks cumul	1314.	4096.24	{2062., 5491.}	215.56	0.440443	newly 5 weeks spec	87.	393.59	{11., 800.}	381.782	0.614361	newly 5 weeks cumul	1559.	5036.55	{2354., 7183.}	226.484	0.442198	newly 51 weeks cumul	1722.	6506.59	{2500., 9503.}	279.556	0.462103
31	newly 3 weeks spec	58.	985.4	{49., 1016.}	1607.1	0.544176	newly 3 weeks cumul	360.	4900.23	{1904., 6571.}	1261.18	0.895471	newly 5 weeks spec	22.	424.05	{0., 428.}	1914.41	0.980471	newly 5 weeks cumul	413.	5990.48	{1982., 6959.}	1350.48	0.866626	newly 52 weeks cumul	442.	6630.92	{1997., 7518.}	1400.21	0.850151
32	newly 3 weeks spec	101.	589.61	{0., 1200.}	601.792	1.97647	newly 3 weeks cumul	549.	8622.18	{3211., 16745.}	1471.62	1.00535	newly 5 weeks spec	30.	256.81	{0., 554.}	894.567	1.81146	newly 5 weeks cumul	633.	9253.28	{3286., 17445.}	1363.	0.967044	newly 49 weeks cumul	682.	9946.42	{3298., 17445.}	1359.66	0.954861
33	newly 3 weeks spec	918.	333.54	{45., 890.}	73.5599	0.731971	newly 3 weeks cumul	3832.	1289.96	{161., 3142.}	69.3784	0.659696	newly 5 weeks spec	479.	235.62	{19., 650.}	81.3236	0.804107	newly 5 weeks cumul	4959.	1802.4	{224., 4535.}	70.9583	0.670705	newly 47 weeks cumul	6261.	3418.27	{492., 10414.}	80.861	0.674421
34	newly 3 weeks spec	843.	1172.24	{34., 3816.}	128.358	0.59308	newly 3 weeks cumul	3518.	7689.94	{890., 16025.}	145.93	0.427314	newly 5 weeks spec	471.	524.63	{2., 2284.}	138.8	1.08851	newly 5 weeks cumul	4638.	8976.93	{1114., 21664.}	121.703	0.36947	newly 42 weeks cumul	6028.	10419.2	{1457., 28352.}	113.414	0.367602
35	newly 3 weeks spec	693.	2847.23	{582., 8179.}	323.632	0.415169	newly 3 weeks cumul	3121.	18516.9	{3226., 63218.}	497.971	0.479162	newly 5 weeks spec	338.	1184.66	{65., 2551.}	301.083	0.553755	newly 5 weeks cumul	3977.	21379.3	{4089., 73142.}	441.267	0.458681	newly 47 weeks cumul	4760.	26106.4	{5561., 85577.}	452.03	0.472617
36	newly 3 weeks spec	635.	3826.07	{450., 6717.}	522.15	0.4597	newly 3 weeks cumul	2904.	11965.4	{2108., 39189.}	330.664	0.435307	newly 5 weeks spec	364.	1851.77	{107., 6826.}	444.777	0.514258	newly 5 weeks cumul	3816.	16606.9	{2684., 59739.}	352.976	0.438761	newly 45 weeks cumul	4844.	23975.8	{3178., 87346.}	411.05	0.47556
37	newly 3 weeks spec	121.	1080.85	{89., 1736.}	807.364	0.653656	newly 3 weeks cumul	634.	5897.2	{533., 5728.}	838.041	0.688223	newly 5 weeks spec	49.	834.51	{4., 1669.}	1634.92	0.895719	newly 5 weeks cumul	759.	7669.07	{741., 8760.}	914.845	0.708646	newly 50 weeks cumul	824.	14705.9	{1107., 53411.}	1686.79	0.858588
38	newly 3 weeks spec	12.	1423.39	{67., 2595.}	11767.6	1.74398	newly 3 weeks cumul	113.	7908.06	{3263., 7957.}	6898.28	1.64351	newly 5 weeks spec	3.	886.26	{2., 3350.}	29460.	1.81141	newly 5 weeks cumul	121.	9880.81	{3944., 13931.}	8065.96	1.70897	newly 50 weeks cumul	126.	15364.9	{3968., 57976.}	12094.4	1.81054
39	newly 3 weeks spec	302.	1339.64	{29., 4350.}	391.371	0.580722	newly 3 weeks cumul	1471.	9396.74	{815., 17386.}	560.749	0.570409	newly 5 weeks spec	115.	506.25	{0., 1197.}	408.217	0.942877	newly 5 weeks cumul	1796.	10672.9	{1124., 23466.}	514.188	0.543214	newly 44 weeks cumul	2002.	12720.9	{1715., 37729.}	548.453	0.54122
40	newly 3 weeks spec	529.	1955.39	{2., 3728.}	316.146	0.868686	newly 3 weeks cumul	2325.	17184.6	{3184., 47414.}	650.923	0.78573	newly 5 weeks spec	275.	604.11	{0., 1111.}	215.058	1.12548	newly 5 weeks cumul	2963.	18796.2	{6496., 47644.}	546.128	0.7341	newly 45 weeks cumul	3697.	20353.6	{6636., 48841.}	462.753	0.680775
41	newly 3 weeks spec	253.	2884.44	{124., 7886.}	1063.04	0.539379	newly 3 weeks cumul	1228.	16261.9	{3776., 52366.}	1225.2	0.770756	newly 5 weeks spec	114.	914.45	{2., 1972.}	793.64	0.972891	newly 5 weeks cumul	1511.	18809.	{3796., 58312.}	1144.8	0.717515	newly 46 weeks cumul	1685.	20178.8	{4188., 60327.}	1097.55	0.703943
42	newly 3 weeks spec	23.	3731.63	{437., 13617.}	16124.5	1.91654	newly 3 weeks cumul	166.	14670.4	{3131., 36618.}	8737.56	1.68669	newly 5 weeks spec	8.	2274.69	{124., 9272.}	28333.6	2.05558	newly 5 weeks cumul	186.	19831.9	{3486., 66642.}	10562.3	1.76791	newly 50 weeks cumul	187.	28019.1	{3601., 94747.}	14883.5	1.89623
43	newly 3 weeks spec	681.	286.08	{0., 620.}	94.1175	1.52638	newly 3 weeks cumul	3118.	5665.15	{151., 15575.}	135.524	0.51971	newly 5 weeks spec	377.	53.76	{0., 124.}	94.9655	2.34952	newly 5 weeks cumul	4043.	5836.64	{160., 15575.}	113.105	0.544826	newly 43 weeks cumul	5018.	5883.54	{161., 15575.}	97.4747	0.57152
44	newly 3 weeks spec	174.	3872.72	{492., 11501.}	2125.7	0.983137	newly 3 weeks cumul	824.	16549.3	{2927., 48456.}	1908.41	1.01456	newly 5 weeks spec	61.	2075.38	{136., 6764.}	3304.1	1.02166	newly 5 weeks cumul	988.	21446.3	{3363., 58394.}	2070.67	1.0361	newly 49 weeks cumul	1084.	26585.4	{3562., 91450.}	2352.53	1.06702
45	newly 3 weeks spec	45.	3047.54	{42., 974.}	6678.53	1.06793	newly 3 weeks cumul	244.	14279.5	{1277., 17359.}	5752.88	1.26001	newly 5 weeks spec	9.	1237.74	{1., 447.}	13680.9	1.38761	newly 5 weeks cumul	276.	17484.1	{1329., 22955.}	6235.22	1.27003	newly 48 weeks cumul	280.	19607.9	{1561., 22956.}	6902.92	1.31245
46	newly 3 weeks spec	223.	2637.49	{33., 6828.}	1114.6	0.806707	newly 3 weeks cumul	1040.	9407.04	{205., 17974.}	827.158	0.752472	newly 5 weeks spec	61.	1535.36	{15., 3348.}	2450.49	1.06105	newly 5 weeks cumul	1223.	12966.2	{282., 30859.}	982.953	0.78773	newly 48 weeks cumul	1322.	17945.3	{394., 46425.}	1278.33	0.836275
47	newly 3 weeks spec	132.	1546.91	{35., 2702.}	1113.57	0.747121	newly 3 weeks cumul	614.	8344.85	{188., 9325.}	1287.47	0.901626	newly 5 weeks spec	43.	754.81	{1., 1620.}	1718.58	1.1106	newly 5 weeks cumul	740.	10166.7	{240., 14371.}	1301.13	0.893553	newly 50 weeks cumul	804.	12517.	{275., 28881.}	1481.82	0.919122
48	newly 3 weeks spec	186.	1356.05	{34., 3033.}	657.382	0.834083	newly 3 weeks cumul	969.	8202.64	{3622., 8727.}	749.591	0.813452	newly 5 weeks spec	49.	909.98	{0., 3577.}	1816.9	1.29293	newly 5 weeks cumul	1115.	10177.6	{4159., 14928.}	815.058	0.839167	newly 50 weeks cumul	1182.	17446.5	{4613., 58814.}	1378.01	0.952063
49	newly 3 weeks spec	8.	1491.53	{16., 1885.}	18553.1	1.71781	newly 3 weeks cumul	68.	6469.85	{155., 7530.}	9415.9	1.6267	newly 5 weeks spec	3.	854.44	{2., 848.}	28398.	1.56605	newly 5 weeks cumul	72.	8462.22	{164., 9664.}	11654.7	1.66643	newly 52 weeks cumul	76.	9645.82	{170., 11076.}	12593.8	1.67152
50	newly 3 weeks spec	733.	740.23	{101., 1380.}	82.0941	0.431421	newly 3 weeks cumul	2944.	8531.47	{3378., 10664.}	195.506	0.325977	newly 5 weeks spec	408.	139.85	{4., 514.}	75.4926	0.981973	newly 5 weeks cumul	3889.	8979.44	{3568., 10792.}	141.302	0.256607	newly 43 weeks cumul	4985.	9127.27	{3586., 10799.}	109.037	0.241149
